# The preliminary analysis of lymphatic flow around the connective tissues surrounding SMA and SpA elucidates patients’ oncological condition in borderline-resectable pancreatic cancer

**DOI:** 10.1186/s12893-024-02398-z

**Published:** 2024-04-13

**Authors:** Hirofumi Akita, Kei Asukai, Yosuke Mukai, Shinichiro Hasegawa, Takeshi Omori, Hiroshi Miyata, Masayuki Ohue, Masato Sakon, Hiroshi Wada, Hidenori Takahashi

**Affiliations:** Department of Gastroenterological Surgery, Osaka International Cancer Institute, 3-1-69 Otemae, Chuo-ku, Osaka, 541-8567 Japan

**Keywords:** Pancreatic cancer, Borderline resectable, Lymphatic flow, Distant metastasis, Arterial connective tissues, Indocyanine green

## Abstract

**Background:**

In pancreatic ductal adenocarcinoma (PDAC), invasion of connective tissues surrounding major arteries is a crucial prognostic factor after radical resection. However, why the connective tissues invasion is associated with poor prognosis is not well understood.

**Materials and methods:**

From 2018 to 2020, 25 patients receiving radical surgery for PDAC in our institute were enrolled. HyperEye Medical System (HEMS) was used to examine lymphatic flow from the connective tissues surrounding SMA and SpA and which lymph nodes ICG accumulated in was examined.

**Results:**

HEMS imaging revealed ICG was transported down to the paraaortic area of the abdominal aorta along SMA. In pancreatic head cancer, 9 paraaortic lymph nodes among 14 (64.3%) were ICG positive, higher positivity than LN#15 (25.0%) or LN#18 (50.0%), indicating lymphatic flow around the SMA was leading directly to the paraaortic lymph nodes. Similarly, in pancreatic body and tail cancer, the percentage of ICG-positive LN #16a2 was very high, as was that of #8a, although that of #7 was only 42.9%.

**Conclusions:**

Our preliminary result indicated that the lymphatic flow along the connective tissues surrounding major arteries could be helpful in understanding metastasis and improving prognosis in BR-A pancreatic cancer.

**Supplementary Information:**

The online version contains supplementary material available at 10.1186/s12893-024-02398-z.

## Introduction

Pancreatic ductal adenocarcinoma (PDAC) is the fourth-leading cause of cancer deaths in the United States, with a 5-year relative survival rate of 8% 1. Surgical resection for localized disease is the only treatment option for a complete cure, but the prognosis after radical resection is still poor, and > 50% of patients develop tumor recurrence at distant or locoregional sites, with an estimated 5-year survival of only 20% 2. One of the reasons for such poor prognosis after radical resection is the high incidence of invasion to extra-pancreatic tissue, including lymphatic vessels and nerve plexuses, leading to distant metastasis. Especially, invasion of connective tissues surrounding major arteries, in which the peripancreatic nerve plexus and lymph vessels exist, is a crucial prognostic factor after radical resection [3–5]. This is why major arterial invasion such as common hepatic artery (CHA) and superior mesenteric artery (SMA) is categorized as borderline resectable state which needs multidisciplinary approach with preoperative therapy for radical resection.

We have also reported that the status of perineural invasion and that of nodal involvement are significant independent prognostic factors in patients with PDAC who are receiving preoperative chemoradiotherapy followed by radical surgery [6]. Furthermore, we have already shown that perineural invasion is significantly associated with not only postoperative local recurrence but with distant metastasis, such as to the liver and non-locoregional lymph nodes [7]. However, why the connective tissues invasion including perineural invasion is associated with postoperative distant metastasis is not well understood. Previous studies have used fluorescence imaging to demonstrate that lymph from the pancreatic head flows into the connective tissues of SMA [8, 9], but how lymphatic flow is running from the SMA connective tissues is still unclear. In this study, we aimed to examine the lymphatic flow from the connective tissues surrounding SMA by using indocyanine green (ICG) fluorescence imaging and clarify why the invasion of connective tissues surrounding SMA is one of risk factors of distant metastasis. Furthermore, we also examined the lymphatic flow from the connective tissues surrounding splenic artery (SpA) and tried to clarify the significance of SpA invasion in pancreatic body and tail cancer.

## Patients and methods

### Enrolled patients

From 2018 to 2020, 25 patients who had undergone radical surgery for pancreatic ductal adenocarcinoma (PDAC) in our institute were enrolled in this study. Patient characteristics are shown in Table 1 and the details of each patient are described in supplementary Table 1. The average age was 62.4 ± 9.1 years old, and 13 patients were male. Twelve patients received pancreaticoduodenectomy for pancreatic head cancer, while 13 patients received distal pancreatectomy for pancreatic body and tail cancer. All patients received D2 lymph node dissection, that included wide sampling of paraaortic lymph nodes. Neoadjuvant chemotherapy and neoadjuvant chemoradiotherapy were performed in 4 and 18 patients respectively, while the other 3 patients received upfront surgery. Pathologically examination was determined to the UICC-TNM classification 8th edition and pathologically lymph node metastasis in the resected specimen was observed in 10 patients and pathologically perineural invasion was observed in four patients. Postoperative recurrence was observed in nine patients and primary recurrence site was as follows; local recurrence in two patients, liver recurrence in five patients, lymph node recurrence in two patients and lung recurrence in two patients.

**Table 1:** The characteristics of enrolled pancreatic cancer patients

Age	62.4 ± 9.1
Sex	
Male/Female	13/12
Location	
Head/Body and Tail	12/13
Preoperative Therapy	
None/NAC/NACRT	3/4/18
pT	
pCR/Tis/T1/T2/T3	1/2/10/9/3
pN	
N0/N1/N2	15/8/2

### Surgical procedure

**Fig. 1:**
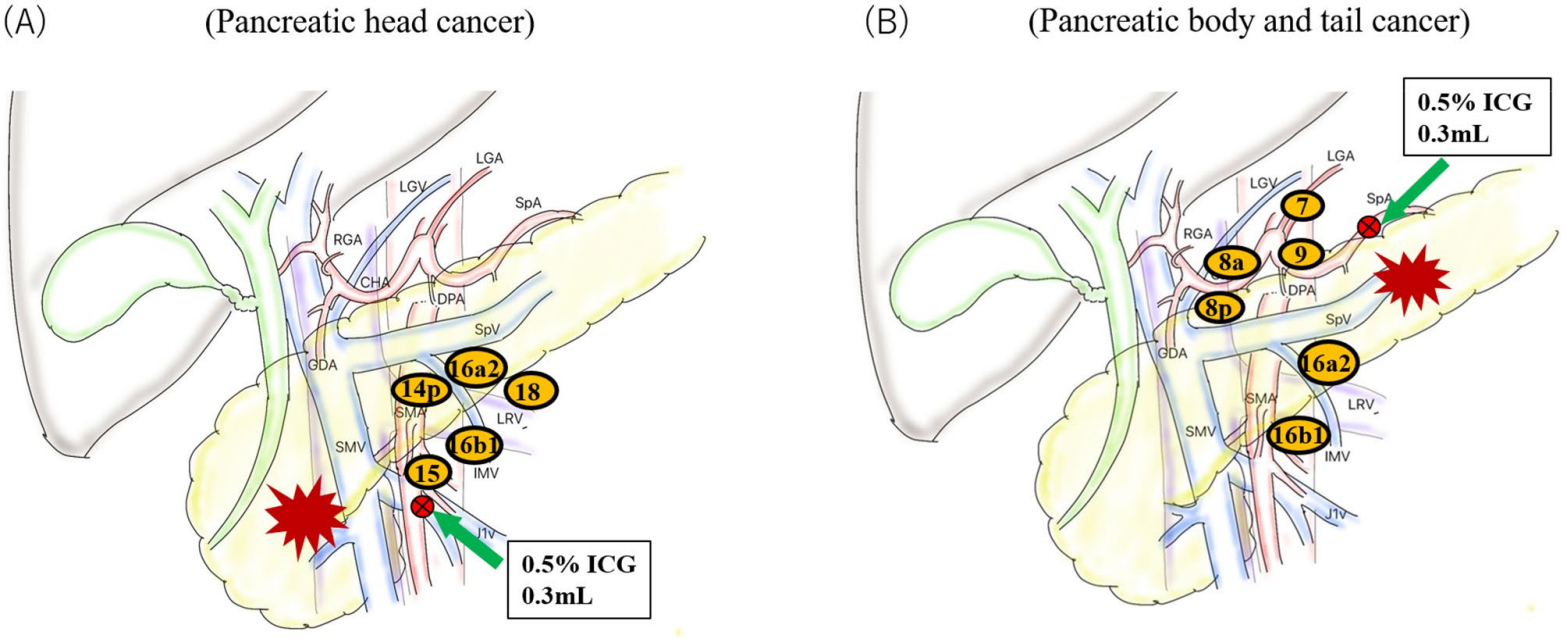
The ICG injection manner according to the location of pancreatic cancer. (**A**) In pancreatic head cancer, ICG was injected into the surface of the nerve plexus in SMA at the level of the middle colic artery bifurcation. Then, the regional lymph nodes along SMA including paraaortic lymph nodes (#16a2 and #16b1) was dissected and examined. (**B**) In pancreatic body and tail cancer, ICG was injected into the surface of the SpA nerve plexus adjacent to the tumor. Then, Then, the regional lymph nodes along SMA including paraaortic lymph nodes (#16a2 and #16b1) was dissected and examined

In pancreatic head cancer, the superior mesenteric vein (SMV) and SMA were exposed at the inferior border of the pancreatic body just after laparotomy. After the SMA sheath was revealed, 0.3 mL of 0.5% ICG was injected into the connective tissues surrounding SMA at the level of the middle colic artery bifurcation (Fig. 1A). Next, we carefully performed Kocher’s mobilization and a wide sampling of paraaortic lymph nodes (#16a2 and #16b1). After that we accomplished pancreaticoduodenectomy by a posterior or mesenteric approach, depending on the location and size of the tumor.

In pancreatic body and tail cancer, we exposed the superior border of the pancreatic body along the SpA, and injected 0.3 mL of 0.5% ICG into the connective tissues surrounding SpA adjacent to the tumor (Fig. 1B). We then performed radical antegrade modular pancreatosplenectomy (RAMPS), including wide sampling of paraaortic lymph nodes (#16a2 and #16b1).

Both in pancreatic head cancer and pancreatic body cancer, we usually dissected affected one-ha-f side of SMA nerve plexus. All patients received D2 lymph node dissection, but #6, #8, #12, #13, #17 lymph nodes in pancreatic head cancer and #10, #11, #18 lymph nodes in pancreatic body-tail cancer were en bloc resected with pancreas, so we couldn’t evaluate the ICG accumulation in these lymph nodes.

### Lymphatic flow analysis

**Fig. 2:**
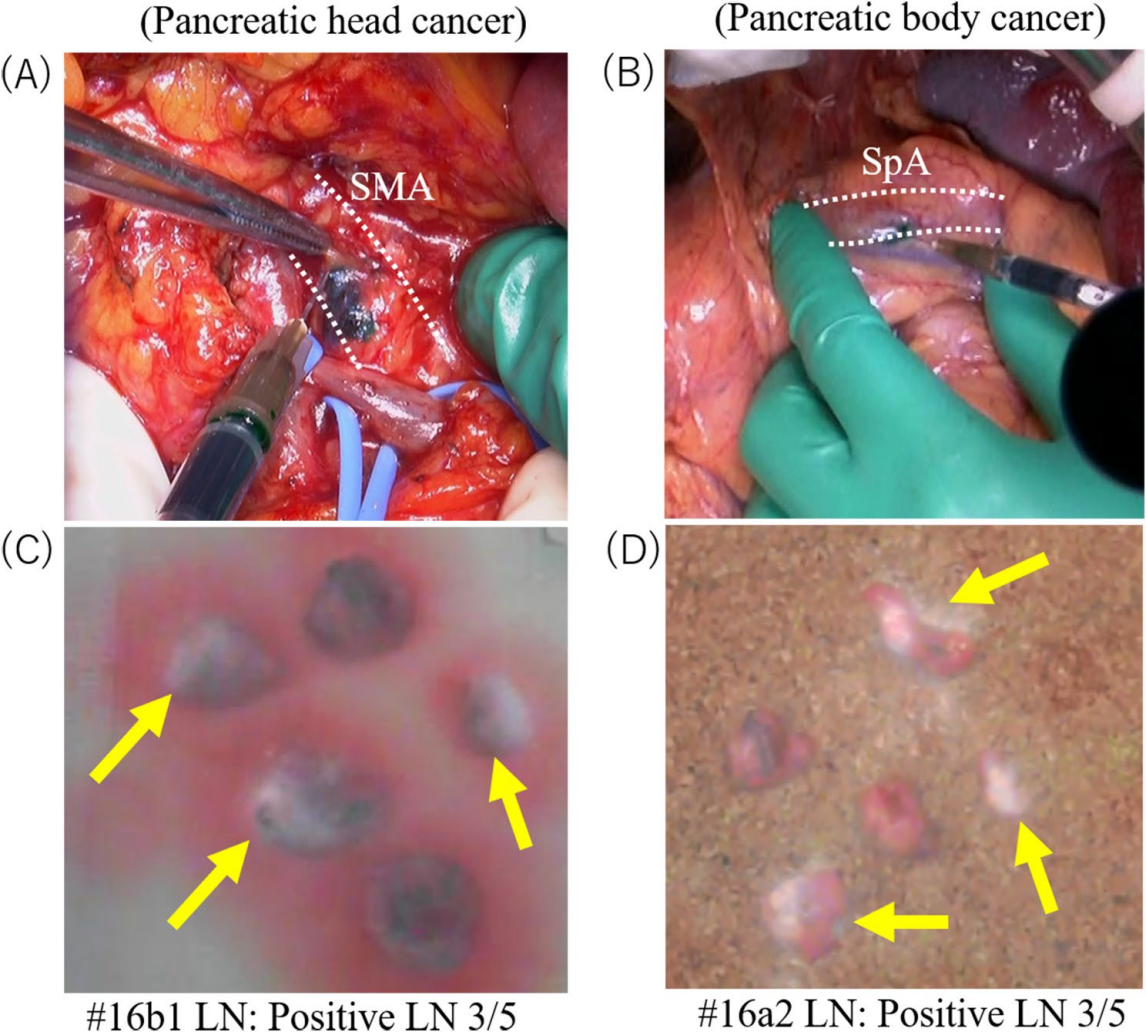
The actual image of ICG injection in a pancreatoduodenectomy (**A**) and a distal pancreatectomy (**B**). The accumulation of ICG in dissected para aortic lymph nodes as detected by HEMS in a pancreaticoduodenectomy (**C**) and in a distal pancreatectomy (**D**)

To examine the lymphatic flow from the connective tissues surrounding SMA and SpA, we used the HyperEye Medical System (HEMS, Mizuho Medical Co. Ltd., Tokyo, Japan), which can visualize ICG-enhanced lymphoid structures and ICG that has accumulated in lymph nodes by detecting the near-infrared fluorescence signal emitted by ICG. In addition, after regional lymph nodes were dissected, whether ICG had accumulated or not in lymph nodes near the SMA or SpA, as shown in Fig. 1, was investigated by HEMS and the percentage of ICG-positive lymph nodes in each region was calculated. The photographs in Fig. 2 show an ICG injection being performed in a pancreatoduodenectomy (Fig. 2A) and a distal pancreatectomy (Fig. 2B). Figure 2 C and 2D show the accumulation of ICG in dissected lymph nodes as detected by HEMS.

### Statistical analysis and ethical issues

All data are expressed as mean ± standard deviation or median and range. Differences in continuous values were evaluated using the Student t-test or Mann–Whitney U test. Categorical data were compared using the Fisher’s exact probability test or Pearson’s chi-squared test, as appropriate. All analyses were performed in IBM SPSS statistics version 21.0 (IBM Japan Business Logistics, Tokyo, Japan), and *P* < 0.05 was considered significant. The statistics expert in our laboratory performed all the statistical analyses.

The study protocol was approved by the Human Ethics Review Committee of Osaka International Cancer Institute (ethical approval number 1,710,059,193). Signed informed consent was obtained from each participant.

## Results

Supplementary Fig. 3 is a moving image showing lymphatic flow, obtained by using HEMS after ICG injection in pancreaticoduodenectomy. ICG-bearing lymph is shown flowing down to the paraaortic area of the abdominal aorta along the connective tissues surrounding SMA. Table 2 shows the percentage of ICG-positive lymph nodes against the total number of dissected lymph nodes in all patients. In pancreatic head cancer, all of LN#14p lymph nodes, which are from the proximal area around the SMA, were ICG positive, indicating that ICG was transported properly within lymphatic vessels and that ICG was stored in lymph nodes. In #16a1 lymph nodes, which were paraaortic lymph nodes near the beginning of the SMA, 9 lymph nodes among 14 (64.3%) were ICG positive and this was higher than the percentage of positive LN#15 (25.0%) or LN#18 (50.0%) nodes, which were near the point of ICG injection, indicating that lymphatic flow around the SMA was leading directly to the paraaortic lymph nodes. Furthermore, the ICG-positive percentages of LN#16b1R and #16b1L were 46.3% and 35.7%, respectively, and also higher than that of #15, indicating that invasion of the SMA by the pancreatic cancer could be contributing to the threat of distant metastasis. Similarly, in pancreatic body and tail cancer, the percentages of ICG-positive #16a2 and #8a nodes were also very high, although that of #7 was only 42.9%, indicating that lymphatic flow into paraaortic lymph nodes was occurring also in the connective tissues surrounding SpA.

**Table 2:** The number of each regional dissected lymph nodes and ICG positive rate

Head (*n* = 12)				Body and tail (*n* = 13)		
LN#	Number of Resected LN	Number of ICG positive LN (%)		LN#	Number of Resected LN	Number of ICG positive LN (%)
14p	5	5 (100)		7	7	3 (42.9)
15	4	1 (25.0)		8a	20	16 (80.0)
16a2	14	9 (64.3)		8p	2	0 (0)
16b1R	41	19 (46.3)		9	2	1 (50.0)
16b1L	42	15 (35.7)		16a2	17	16 (94.1)
18	8	4 (50.0)		16b1L	10	0 (0)

Table 3 indicates the percentage of patients with ICG-positive lymph nodes in each lymph node lesion. In patients with pancreatic-head cancer, paraaortic lymph nodes (#16b1R) were detected in all patients, and ICG-positive nodes were detected in 10 patients (83.3%). Furthermore, in patients with pancreatic body and tail cancer, paraaortic lymph nodes (#16a2) were detected in 11 patients (84.6%), and all patients showed ICG positivity. These data indicate that pancreatic cancer was easily infiltrating paraaortic tissue along connective tissues surrounding major arteries, leading to distant metastasis.

**Table 3:** The percentage of patients with ICG-positive lymph nodes in each lymph node lesion

Head (*n* = 12)				Body and tail (*n* = 13)		
LN#	Number of detected patients	Number of ICG positive patients(%)		LN#	Number of detected patients	Number of ICG positive patients(%)
14p	2	2 (100)		7	4	3 (75.0)
15	2	1 (50.0)		8a	11	9 (81.8)
16a2	9	7 (77.8)		8p	2	0 (0)
16b1R	12	10 (83.3)		9	2	1 (50.0)
16b1L	11	6 (55.6)		16a2	11	11 (100)
18	4	2 (50.0)		16b1L	4	0 (0)

## Discussion

In borderline-resectable pancreatic cancer, the surgery-first approach is not curative. Most cases result in tumor relapse because of both the high risk of positive margins and the high incidence of early distant recurrence, including non-regional lymph node metastasis. Thus, the National Comprehensive Cancer Network (NCCN) guideline recommends preoperative chemotherapy with or without radiotherapy. We have also reported the prognostic benefit of preoperative chemoradiotherapy for patients with resectable and borderline-resectable pancreatic cancer [10]. However, especially in borderline-resectable cases with SMA abutment, the surgical outcome was unsatisfactory, mainly because of distant metastasis, although local control of the cancer was relatively good, probably due to the effect of radiation therapy [7]. Thus, to improve the prognosis of patients with pancreatic cancer with SMA abutment, it is important to understand why SMA invasion leads to distant metastasis.

We proved in this study that there are some lymphatic vessels in the connective tissues surrounding SMA. Previously, Xu et al., using normal autopsy specimens, revealed that lymphatics and capillaries are present in the mesopancreatic root, located between the uncinate process of the pancreas and the superior mesenteric vessels [11]. They also revealed that intra-mesopancreatic nerves, lymph nodes, lymphangions, and fascia fibers along the SMA were infiltrated by cancer cells in specimens of unresectable pancreatic cancer. Furthermore, Cheng et al. described the invasion of lymphatic vessels along the SMA as activating tumor-induced lymphangiogenesis, resulting in the development of metastatic tumors [12]. Our results also show that tumor cells could easily move into the general circulation within a few minutes when tumor cells invaded the connective tissues surrounding SMA. This indicates that the clinical condition in borderline-resectable pancreatic cancer with initial artery abutment (BR-A) was completely different from borderline-resectable pancreatic cancer with portal vein invasion (BR-PV), so we should consider a distinct treatment strategy for BR-A, separately from BR-PV. In surgical procedures, the mesenteric approach, one of six approaches to the superior mesenteric artery [13], was reported to be suitable for BR-A pancreatic cancer with respect to the early judgement of resectability and a sufficient peripancreatic margin around the SMA. However, Hirono et al. reported that the mesenteric approach did not provide significant prognostic advantages for patients with borderline-resectable pancreatic cancer, although it could yield prognostic benefits to patients with resectable pancreatic cancer in the form of lower local recurrence rates [14]. Taking into consideration our result that cancer cells easily migrated into the systemic circulation when pancreatic cancer invaded the connective tissues surrounding SMA, it is not surgical techniques but an effective multimodal approach including powerful preoperative chemotherapy that is essential for the prognostic improvement of BR-A pancreatic cancer patients.

In this study, we injected ICG along the connective tissues surrounding SpA in patients with pancreatic body and tail cancer. The injected ICG was transported in lymphatic vessels of the connective tissues surrounding SpA into paraaortic lymph nodes; this is very similar to the observed ICG transport in the connective tissues of SMA in patients with pancreatic head cancer. Recent reports have described pancreatic body and tail cancer with SpA involvement showing poor prognosis after radical resection [15–17]. Similarly, we have reported that SpA involvement is a poor prognostic indicator after radical resection in patients with pancreatic body and tail cancer who receive preoperative chemoradiotherapy [18]. Interestingly, the incidence of distant recurrence was significantly high in patients with SpA involvement, and this was very similar to the results in patients with SMA abutment, potentially indicating that pancreatic body and tail cancer with SpA involvement should be treated as borderline resectable like pancreatic head cancer with CHA and SMA invasion.

Of course, this study was just exploratory research and the interpretation of the results is limited by its small sample size, so we have to elucidate the lymphatic flow in the connective tissues surrounding SMA more definitively in a larger number of patients. However, our results could partially explain why BR-A pancreatic cancer shows a high incidence of distant metastasis after radical resection, and we believe that our results could help to establish a more effective treatment strategy in BR-A pancreatic cancer.

### Electronic supplementary material

Below is the link to the electronic supplementary material.

Supplementary Material 1

Supplementary Material 2

Supplementary Material 3

Supplementary Material 4

## Data Availability

The datasets used and/or analysed during the current study are available from the corresponding author on reasonable request.
