# Supplementary material for: The preliminary analysis of lymphatic flow around the connective tissues surrounding SMA and SpA elucidates patients’ oncological condition in borderline-resectable pancreatic cancer
Source: BMC Surg. 2024 Apr 13;24:107. doi: 10.1186/s12893-024-02398-z (PMC11015602; doi:10.1186/s12893-024-02398-z)
Supplement: Supplementary file 4 — Supplementary Material 4 [file 12893_2024_2398_MOESM4_ESM.docx]

Supplementary Figure3

The moving image showing lymphatic flow, obtained by using HEMS after ICG injection in pancreaticoduodenectom
